# Doubly Disadvantaged? Bullying Experiences among Disabled Children and Young People in England

**DOI:** 10.1177/0038038515574813

**Published:** 2015-04-28

**Authors:** Stella Chatzitheochari, Samantha Parsons, Lucinda Platt

**Affiliations:** University of Warwick, UK; Institute of Education, University College London, UK; London School of Economics and Political Science, UK

**Keywords:** bullying, children, disability, Longitudinal Study of Young People in England, Millennium Cohort Study, school, young people

## Abstract

Bullying among school-aged children and adolescents is recognised as an important social problem, and the adverse consequences for victims are well established. However, despite growing interest in the socio-demographic profile of victims, there is limited evidence on the relationship between bullying victimisation and childhood disability. This article enhances our understanding of bullying experiences among disabled children in both early and later childhood, drawing on nationally representative longitudinal data from the Millennium Cohort Study and the Longitudinal Study of Young People in England. We model the association of disability measured in two different ways with the probability of being bullied at ages seven and 15, controlling for a wide range of known risk factors that vary with childhood disability. Results reveal an independent association of disability with bullying victimisation, suggesting a potential pathway to cumulative disability-related disadvantage, and drawing attention to the school as a site of reproduction of social inequalities.

## Introduction

Conceptualised as a repetitive and intentionally harmful form of aggression that involves a power imbalance between the victim and the perpetrator(s) (Olweus, 2003), bullying is increasingly identified as a significant social problem across a large number of countries. A recent government survey in England reported that one in two children aged 8–16 years old worry about school bullying, while 18 per cent admitted they had been bullied regularly at school last month (Chamberlain et al., 2010). Prevalence rates are also high in other countries although they vary considerably with the age of the children examined and the measurement of bullying victimisation employed (Stassen Berger, 2007). The phenomenon has recently attracted considerable policy attention, and a combination of proactive and reactive strategies has been adopted by English schools to lessen its occurrence (Department for Children, Schools and Families, 2008; Department for Education, 2011).

Bullying has detrimental consequences. Aside from its immediate health and psychological impacts (Nansel et al., 2001; Rigby, 2000), being a victim is a predictor of low self-esteem, anxiety and depression during adulthood, and has a negative impact on subsequent socio-economic attainment (Arseneault et al., 2010; Takizawa et al., 2014; Wolke et al., 2013). Previous research has also established links with eating disorders, truancy and suicidal ideations (Nansel et al., 2001; Rigby and Slee, 1993). These findings suggest that bullying may constitute an important pathway through which social inequalities across a range of domains are reproduced, underlining the importance of identifying those who experience a higher risk of being bullied in early childhood and adolescence.

Childhood disability has been largely overlooked in the growing body of quantitative research focusing on risk factors for bullying victimisation and the socio-economic profile of victims. Additionally, the majority of existing research is embedded in medical rather than social models of disability, failing to consider negative representations of disability as ‘difference’ and the potential role of school processes in facilitating the conditions within which bullying of disabled children is likely to occur (Holt, 2004). This is despite the fact that qualitative research from disability scholars suggests that bullying is a pervasive experience in disabled children’s daily lives (Connors and Stalker, 2002; Watson et al., 1999). Indeed, bullying can be represented as one of the means by which children with impairments or particular needs become ‘disabled’. According to the social model of disability, bullying constitutes a ‘barrier to being’ that affects sense of self and well-being, thus playing an important role in the process that has been termed ‘psycho-emotional disablism’ (Connors and Stalker, 2007; Thomas, 1999). At the same time, previous research has found that perceived peer *support* constitutes an important coping mechanism for disabled children, engendering better social and academic adjustment (Wallander and Varni, 1998). Consequently, bullying can be expected to undermine self-efficacy (Bandura, 1997) and to contribute to the adverse psychological and social outcomes commonly found among individuals who have experienced childhood disability (Janus, 2009; Priestley, 2003; Wells et al., 2003), thereby reinforcing the social disparities that render disability a crucial marker of social inequality (Priestley, 2003). It is therefore essential to develop a sociological understanding of how bullying renders disabled children doubly disadvantaged within the school system and to integrate that understanding systematically into life-course research on inequalities linked to disability (Janus, 2009; Link and Phelan, 2001; Powell, 2003).

In order to establish the extent to which bullying specifically does render disabled children liable to negative long-term consequences, it is necessary first to provide representative evidence on the victimisation of disabled children and young people, using a comprehensive, social understanding of disability. This study, therefore, aims to establish whether the relationship between childhood disability and the risk of being bullied suggested by qualitative research exists when taking account of other risk factors such as the greater socio-economic disadvantage typically faced by both disabled children and bullying victims (Blackburn et al., 2010; Dowling and Dolan, 2001). Drawing on longitudinal large-scale data from the Millennium Cohort Study and the Longitudinal Study of Young People in England, we examine bullying experiences at ages seven and 15 in contemporary England. These datasets allow us to advance understanding of experiences of disabled children and young people in a number of ways. First, we distinguish two overarching measures of ‘disability’, special educational needs and long-term limiting illness. Unlike extant studies embedded in the medical model of disability linking specific conditions with bullying, our study adopts a social model of disability, locating disability in the ways in which physical and mental impairments become constructed as ‘disabling’. Acknowledging that different constructions of disability may have different implications for victimisation, we investigate whether enhanced risks are associated with either of these measures to determine whether there are differentiated experiences of bullying with childhood disability. Second, we provide a nationally representative picture at two age points, investigating whether risks associated with disability are consistent across childhood and adolescence. Third, our analysis moves beyond cross-sectional designs that do not establish a temporal order between risk factors and bullying experiences. Fourth, we consider a wider range of known risk factors than existing studies, providing a more robust test of the relationship of interest.

The following section presents our theoretical framework and an empirical literature review on bullying and disability. We then discuss data and methods, followed by the presentation and discussion of analyses. We conclude by discussing the implications of our findings.

## Childhood Disability and Bullying Victimisation: Theoretical Framework and Literature Review

Recent years have witnessed an increased interest in the study of bullying among children and adolescents. Recognition of the long-term negative consequences of childhood victimisation has led to a substantial strand of research on the identification of the socio-economic and behavioural characteristics of bullying victims (Griffin and Gross, 2004). Despite substantial variation in study design and operationalisation of bullying across different studies, the consensus of accepted knowledge on the demographic and socio-economic profile of victims has advanced significantly over the last decade. A number of studies has demonstrated that gender, age, appearance, school achievement, family circumstances, parenting style, socio-economic status (SES) and ethnicity may exert a significant influence on the risk of being bullied (Fox and Farrow, 2009; Wolke and Skew, 2012; Wolke et al., 2001).

Bullying itself has been differently conceived in research, with a variety of methods employed to measure bullying experiences, ranging from observational studies and teacher reports to questionnaires asking respondents how frequently they have been subjected to certain forms of aggression. However, fundamental to the conceptualisation and operationalisation of bullying is recognition of its relational nature, whereby the perpetrator uses victimisation to substantiate or reinforce unequal power relationships and maintain a social hierarchy within a particular group or network (Faris and Felmlee, 2014). Hence, self-report can be regarded as the most appropriate method of assessing bullying experiences (Olweus, 2003; Rigby and Slee, 1993; Woods and Wolke, 2004), as it prioritises the victim’s perception of their experience. Moreover, it allows researchers to distinguish between different types of bullying, namely physical and relational bullying. Physical bullying refers to direct forms of violence such as hitting, kicking and so on, while relational bullying refers to less obvious forms of aggression aiming to harm relationships, such as excluding a classmate from a group and spreading humiliating gossip (Smith et al., 2002). This analytical distinction is particularly important when examining the occurrence of bullying in adolescence, since it is characterised by a higher frequency of strategic relational bullying compared to childhood when physical bullying is the predominant form of aggression (Griffin and Gross, 2004).

Currently, the majority of empirical research comes from the discipline of psychology. As a result, bullying has been widely conceptualised in terms of individual and family pathology, with little attention to social processes and mechanisms that put particular groups at risk (Søndergaard, 2012). A more insightful approach from within sociology focuses on asymmetric power relationships and the role of bullying for social climbing (Faris and Felmlee, 2014). According to this perspective, bullying is used to attain social status in the school network hierarchy, with weak and vulnerable populations comprising ‘easy targets’ and bearing the brunt of abuse. Disabled children are often regarded among such vulnerable groups, occupying marginal positions in school settings (Faris and Felmlee, 2014). This is confirmed by earlier research on the attitudes of non-disabled students towards their disabled peers, which suggests low levels of popularity and that disability often interferes in processes of friendship formation (Hodkinson, 2007). The social relational model of disability suggests that such negative perceptions result from structural processes that promote normative assumptions about appropriate childhood development and labelling of children with impairments or needs as ‘others’ through negative representations of difference (Fine and Asch, 1988; Hollomotz, 2012; Holt, 2004; Oliver, 1992; Powell, 2003; Watson et al., 1999).

This claim is particularly relevant for students formally identified as having Special Educational Needs (SEN), especially those with a statement of needs, which outlines the specialist support they require beyond existing provision. Disability scholars have problematised the SEN label in English mainstream schools, drawing attention to the ways it produces beliefs about normality and difference and stigmatises students with learning needs (Holt, 2004; Powell, 2003). For example, while acknowledging variation in practices between schools, Holt’s (2004) qualitative study of SEN primary school students underlines how boundaries between disabled and non-disabled students are reproduced through within-school segregation. This focus on SEN and on the effect of labelling aligns with Goffman’s original conceptualisation of stigma that stressed the importance of visibility of stigmatised characteristics to others (Goffman, 1963; Link and Phelan, 2001; Socall and Holtgraves, 1992). However, while the visibility of SEN (with statement) may put labelled students at the highest risk of victimisation in the school context, it is likely that disabled students who are not institutionalised in this way will also be perceived as different and subject to ‘othering’. This is because their impairments may interfere with their social interactions with other students or be linked with different aspects of physical appearance, an attribute linked to peer popularity and school aggression across different contexts (Faris and Felmlee, 2014; Goffman, 1963; Holt, 2004).

The relationship between childhood disability and bullying victimisation has been surprisingly under-researched and these theoretical claims have not been systematically tested. One of the main weaknesses of previous studies is the lack of consideration of other risk factors in order to ascertain whether bullying experiences of disabled children are indeed linked to their disability or to other characteristics that may also affect their popularity and position in the school hierarchy. For example, the majority of studies reporting an increased risk of being bullied among students with special learning needs (Baumeister et al., 2008; Mishna, 2003; Nabuzoka and Smith, 1993; Norwich and Kelly, 2004; Rose et al., 2009; Thompson et al., 1994; Whitney et al., 1992) have not used large-scale samples and were unable to establish whether the risk was due to confounding factors such as low SES (Blackburn et al., 2010; Dowling and Dolan, 2001; Parsons and Platt, 2013) or the poorer educational performance that is common among children with learning needs.

A number of small-scale studies have also examined whether children suffering from specific chronic physical and psychological conditions are more likely to be bullied, focusing on single conditions such as cerebral palsy or diabetes. The majority of findings report a higher risk, but some studies do not find any differences between disabled and non-disabled children, even in cases of observable conditions (Rose et al., 2010). Aside from their small sample size and limited generalisability, an additional shortcoming of these studies is a medical understanding of disability that links victimisation to the condition itself and does not acknowledge social constructions of disability and the resulting asymmetric power relationships in the school setting (Oliver, 1992).

Clearer evidence has been provided from school-based studies covering particular areas. For example, using cross-national data from the Health Behaviour in School-Aged Children survey, Sentenac et al. (2013) reported a strong association between disability and bullying victimisation among adolescents in 11 European countries. Similar findings have been provided by Sweeting and West (2001), focusing on 11-year-olds in West Scotland.

While these studies present a prima facie case for a relationship between bullying and childhood disability, nationally representative analyses are limited to a few recent studies focusing on the USA (Son et al., 2012; Turner et al., 2011). There is therefore a need for further quantitative studies that consider different overarching types of disability and scrutinise a wider range of factors in order to understand better the risks faced by different groups of disabled children as well as the mechanisms leading to bullying victimisation. This article responds to this challenge and provides a longitudinal analysis of the relationship between childhood disability and the risk of being bullied in contemporary England.

## Data, Methods and Measures

### Datasets

We analyse nationally representative longitudinal data from the Millennium Cohort Study (MCS) (University of London, 2012a, 2012b, 2012c, 2012d) and the Longitudinal Study of Young People in England (LSYPE) (Department for Education and National Centre for Social Research, 2012). These datasets provide sufficiently large subsamples of disabled children and young people, allowing us to rectify the recognised lack of reliable quantitative analyses of childhood disability in the UK (Blackburn et al., 2010).

MCS follows approximately 19,000 children born in 2000–2001. Five surveys have been carried out so far – at age nine months, three, five, seven and 11 years. LSYPE is a panel survey of around 16,000 young people born in 1989–1990, sampled from schools in England and interviewed annually between 2004 (at age 13/14) and 2010 (at age 19/20). For comparison with the LSYPE, the MCS sample is restricted to children living in England. As our bullying outcomes were measured at ages seven and 15, we focus on a four-wave longitudinal sample of 7342 children (MCS) and a three-wave longitudinal sample of 12,144 young people (LSYPE).

All analyses are adjusted for the complex sampling design of both surveys and for non-response. We investigated patterns of attrition and found no evidence for an increased risk of dropping out among disabled respondents, which could potentially have biased our estimates.

### Bullying Measures

Bullying victimisation on MCS was measured with the question ‘how often do other children bully you?’, with three available response options: never; some of the time; and all of the time. We define those respondents who responded ‘all of the time’ as victims. Given the response options, children who experienced isolated bullying incidents are likely to be included in the ‘some of the time’ category. We therefore adopted a stringent threshold to capture repetition, which is a key element across different bullying definitions (Olweus, 2003).

LSYPE respondents were asked five questions on whether and how often they were subjected to different forms of aggression in the last year. Frequency was measured with a six-item response scale running from ‘every day’ to ‘less often than once a month’, with an additional measure for ‘it varies’. We constructed a physical bullying category if the respondent experienced one or more of the following ‘once every two weeks’ or more often: (1) being made to hand over money and possessions; (2) receiving violence threats; and (3) being a victim of physical violence. The relational bullying category was constructed by combining responses referring to: (1) being excluded by a group of friends; and (2) being called names, including by text or email, using the same frequency threshold. It should be noted that respondents were not asked directly about ‘bullying’ but about specific acts. Hence, if there were experiences they regarded as bullying, but which they were not asked about, they would not be counted in our analysis.

LSYPE bullying items mentioned the word ‘students’, while the MCS question was placed among other school-related items in the self-completion questionnaire. We are therefore confident that our measures refer to school bullying. However, our measures are not directly comparable across the two surveys and hence we are unable to compare prevalence in childhood and adolescence directly.

### Disability Measures

We distinguish between limiting long-standing physical or mental health conditions and learning needs identified as SEN. Both surveys collected information on having a statement of SEN, and pupils with statements were more likely to face multiple learning needs than those reported to have SEN but no statement. SEN statement is therefore an indicator of severity of learning needs and, according to our theoretical framework, a label that renders pupils ‘visible’ and thus more vulnerable to peer harassment in the school setting than other disabled groups. Long-standing limiting illness (LSLI) refers to a condition or impairment lasting over 12 months that limits daily/school activities. The measure is in line with the equalities legislation that identifies activity limitation and length of condition as defining characteristics of disability.

MCS collected information on cohort members’ LSLI at ages three, five and seven. Eleven per cent of the sample had LSLI in one or more survey waves. SEN was measured at age seven, with approximately 17 per cent of children identified as SEN, of whom 4 per cent had a statement. In LSYPE, LSLI was covered in Wave one, while SEN-related questions were asked in Waves one and two. Six per cent of young people in the sample had LSLI, whereas 17 per cent were identified as currently having SEN, of whom 5 per cent had a statement.

### Independent Variables

Both surveys collected rich information on respondents’ family and socio-demographic circumstances, enabling us to consider a wide range of factors likely to influence the risk of being bullied. The longitudinal nature of the studies allows us to incorporate temporal ordering between risk factors and bullying outcomes.

We control for children’s demographic characteristics, namely gender, age for school year (season born) and ethnicity. Previous research suggests that boys face an overall higher risk of being bullied, while girls are more likely to be subjected to relational forms of bullying during adolescence (Stassen Berger, 2007). Those young for their school year may face a higher risk as a result of being or appearing physically weaker than their classmates or because of lower academic attainment (Crawford et al., 2013). There is some evidence that both bullying and childhood disability are patterned by ethnic background (Blackburn et al., 2010; Vervoot et al., 2010). However, it is difficult to hypothesise a specific association between the two as previous findings are not clear-cut (Tippett et al., 2013). Since status hierarchies will be influenced by the ethnic profile of students in each school, exploratory models also controlled for ethnic composition. However, there was no significant association and the variable was dropped.

Socio-economic disadvantage has also been found to be a predictor of being bullied, though results are not entirely consistent (Faris and Felmlee, 2014; Wolke and Skew, 2012). We consider multiple dimensions of SES measured in the first wave of both surveys, namely housing tenure, parental educational qualifications, parental worklessness and whether the child lives in a lone parent family.

Additionally, we examine the effects of family size and maternal mental health/disability, which have been linked to disability as well as behavioural and bullying outcomes but have been largely neglected in previous bullying research (Turner et al., 2011). Parents of disabled children are more likely to experience psychological distress, with subsequent implications for parenting style and child–parent relations (Breslau et al., 1982). Parenting has been identified as an important mediator of bullying risk (Wolke and Skew, 2012). In the MCS analysis, we control for closeness between mother and child, assessed by the mother at age five. We also consider parenting style, focusing on the effect of frequent use of harsh discipline measures. Although LSYPE does not include identical parenting measures, we exploit information on frequency of arguments between main parent and child measured at Wave one as a proxy for closeness/conflict.

As bullying may be focused on the intellectual/academic attainment of disabled children we employ controls for cognitive ability, namely the Naming Vocabulary score from the British Ability Scales Second Edition (BAS II) (Elliott, 1996) that was administered at age five in MCS, and Key Stage 2 (age 11) overall attainment score of LSYPE respondents. Even though disabled children tend to have lower cognitive/KS2 scores than other children, there is substantial overlap, rendering this a relevant control. MCS analyses additionally control for the effects of being short and/or overweight (Fox and Farrow, 2009), by controlling for weight (Body Mass Index) and height. LSYPE did not collect information on these domains.

The influence of contextual characteristics such as school size and proportion of students with a SEN statement was also examined. We expected that SEN students would be more marginalised and vulnerable in schools with a lower SEN statement percentage (Faris and Felmlee, 2014). We did not find significant variation across these contextual variables, and excluded them from our final models.

Variance inflation factors (VIFs) were calculated to ensure that the large set of variables did not raise collinearity issues. All of the VIFs were small. We therefore retained all independent variables in the full models, since they were theoretically expected to be important.

### Analytical Technique

Logistic regression models were estimated to examine whether there was an independent relationship between disability and bullying, net of other risk factors. The outcome is *being bullied* and the binary response is *yes/no*. Our models estimate the relative effect of disability status and other variables on the probability of being bullied at ages seven and 15. We report unadjusted and adjusted log odds, and we also present predicted probabilities of being bullied by disability status at average levels of all other risk factors to illustrate better the magnitude of the association between being disabled and being bullied.

## Results

This section presents descriptive statistics on independent variables by disability status, followed by the results of multivariate analyses. For reasons of parsimony, we concentrate on the relationship of interest and only briefly discuss the associations of bullying victimisation with the other variables in the adjusted models.^1^

Tables 1 and 2 provide information on family and child characteristics by disability status at ages seven and 15 respectively. Consistent with previous findings, disabled children and young people are more disadvantaged than their non-disabled peers across all four socio-economic dimensions examined, and those with SEN statement are the most deprived. Focusing on MCS (Table 1), we also observe that disabled children appear more likely to be obese/overweight and to have lower levels of cognitive abilities compared to non-disabled children. In general, we find similar patterns for all disabled groups across the majority of independent variables, with the exception of maternal report about feeling ‘extremely’ close to the child, where there is a significant difference between SEN statement and all other groups. This could indicate communicative competence and social interaction difficulties that have been previously linked with special learning needs and with the victimisation of SEN children in school settings (Mishna, 2003).

**Table 1.** Descriptive statistics of family and child characteristics by disability status (MCS), column %/mean values.

	All	No SEN	SEN	Statement	No LSLI	LSLI
**Family characteristics**						
*Housing*						
Home owner	63.1	66.1	51.4	41.3	64.3	51.6
Social housing	23.3	21.0	31.9	40.8	22.4	32.2
Private rented	8.2	7.8	10.3	10.0	7.9	10.7
*Education*						
Degree or higher	43.1	45.5	33.5	26.4	43.8	37.1
NVQ3 (A levels)	15.6	15.7	15.1	16.2	15.9	13.3
NVQ2 (O levels)	25.2	24.3	30.6	27.2	24.9	27.8
NVQ1 (Level 1/CSE)	5.9	5.3	8.1	10.7	5.8	7.3
No qualifications	10.1	9.2	12.7	19.5	9.6	14.5
*Household type*						
Single parent	13.1	12.2	16.8	18.7	12.4	19.3
Workless household	16.2	14.3	23.7	29.8	15.2	25.5
Mean no. of children (standard error)	2.5	2.5	2.6	2.6	2.5	2.5
	(.02)	(.02)	(.04)	(.07)	(.02)	(.04)
Mean mother malaise score (standard error)	1.6	1.5	1.9	2.1	1.6	2.1
	(.03)	(.03)	(.06)	(.11)	(.03)	(.07)
Mean discipline score (standard error)	17.9	17.7	18.4	18.8	17.8	18.3
	(.06)	(.06)	(.14)	(.30)	(.07)	(.16)
‘Extremely’ close with child	69.3	69.9	68.0	62.4	68.9	71.4
**Child characteristics**						
Male	50.8	48.0	61.2	71.9	49.9	58.2
Minority ethnic group	15.4	16.0	11.6	17.1	15.4	16.6
Mean height (cms) (standard error)	123.7	123.9	123.1	122.8	123.8	123.3
	(.09)	(.09)	(.19)	(.38)	(.09)	(.27)
BMI overweight	14.2	14.3	13.3	15.9	14.2	13.9
BMI obese	5.7	5.2	7.3	9.4	5.2	9.6
*Season born*						
Autumn	28.6	29.6	23.9	24.1	28.7	28.1
Winter	26.4	26.5	25.5	26.9	26.1	28.2
Spring	18.5	18.5	16.7	22.5	18.5	18.2
Summer	26.5	25.3	34.0	26.4	26.7	25.6
Mean BAS naming vocabulary score (standard error)	108.1	109.9	101.7	92.7	108.6	103.7
	(.42)	(.42)	(.71)	(1.54)	(.40)	(.90)

*Note*: All values are group percentages except where indicated as mean and standard error. All statistics adjusted for complex survey design and non-response.

**Table 2.** Descriptive statistics of family and child characteristics by disability status (LSYPE), column %/mean values.

	All	No SEN	SEN	Statement	No LSLI	LSLI
**Family characteristics**						
*Housing*						
Home owner	72.2	74.2	63.2	53.9	73.1	59.4
Social housing	22.1	20.1	31.4	39.2	21.2	34.5
Private rented	5.7	5.7	5.5	6.9	5.7	6.1
*Education*						
Degree or higher	17.3	18.1	15.9	17.9	17.7	11.8
Below degree	15.4	15.9	14.2	10.8	15.3	16.8
A Level	17.7	17.9	17.1	16.0	17.7	17.6
GCSE A–C	27.1	27.2	25.6	28.2	27.3	24.6
Level 1 (and below)	6.7	6.2	9.1	10.6	6.4	10.7
Other quals	1.3	1.2	2.1	1.8	1.3	1.7
No qualifications	14.4	13.5	18.0	23.0	14.3	16.9
*Household type*						
Single parent	23.7	22.6	29.6	33.0	23.3	30.4
Workless household	14.4	12.7	21.0	30.6	13.4	28.3
Mean no. of children (standard error)	2.2	2.2	2.3	2.3	2.2	2.3
	(.01)	(.01)	(.04)	(.06)	(.01)	(.05)
Mother disabled	13.0	12.0	19.7	19.0	12.1	24.5
Arguments most days/most of the time	37.5	35.9	46.1	49.8	36.9	47.9
**Child characteristics**						
Male	50.7	48.6	61.1	68.2	50.3	55.6
Minority ethnic group	13.4	14.3	7.1	8.3	13.8	8.8
*Season born*						
Autumn	24.4	24.7	21.7	22.7	24.4	23.7
Winter	23.8	24.0	21.6	22.7	24.1	20.0
Spring	25.4	25.2	27.4	25.7	25.2	27.5
Summer	26.5	26.1	29.4	28.9	26.3	28.7
Mean Key Stage 2 score (standard error)	27.1	27.8	23.6	20.8	27.3	23.7
	(.08)	(.06)	(.17)	(.42)	(.07)	(.33)

*Note*: All values are group percentages except where indicated as mean and standard error. All statistics adjusted for complex-survey design and non-response.

Table 2 reveals similar differences in cognitive ability at age 15. Additionally, we observe that disabled young people are more likely to engage frequently in arguments with their mother and to have a disabled mother. Again, these are in line with our expectations from the literature, which has shown that parental disability is not only a risk for bullying but is also more prevalent among the parents of disabled children and young people (Blackburn et al., 2010). Moreover, enhanced parent–child conflict has been linked both to the more difficult socio-economic circumstances disabled children and young people face, as well as to the specific difficulties of intra-personal communication that can arise in parenting a disabled child (Howe, 2006). Overall, Tables 1 and 2 confirm that family circumstances, socio-economic disadvantage and cognitive ability vary by disability status in early childhood and adolescence. We now examine the extent to which these factors are implicated in the victimisation of disabled children and adolescents.

Table 3 presents unadjusted and adjusted coefficients from logistic regressions predicting the probability of being bullied at ages seven and 15, focusing on disability status. Other independent variables had coefficients largely in the expected direction. However, family structure, child height and number of siblings were not associated with bullying risks net of other factors at age seven, while mother’s malaise score (Rutter et al., 1970) was in the opposite direction to that expected. At age 15, SES measures were not significantly associated with bullying, but all other covariates were in the expected direction.

**Table 3.** Probability of experiencing bullying at age seven and age 15 by disability status, estimates from logistic regression models.

***MCS (age 7)***	Bullied ‘all’ of the time			
	Unadjusted: Coeff (SE)		With controls+: Coeff (SE)	
*SEN status (ref.=no SEN)*				
Has SEN	1.04 (0.13)***		0.69 (0.14)***	
Has statement of needs	1.21 (0.21)***		0.65 (0.24)**	
*LSLI status (ref.=no LSLI)*				
Has LSLI	0.60 (0.13)***		0.39 (0.15)**	
***LSYPE (age 15)***	Physical bullying		Relational bullying	
	Unadjusted: Coeff (SE)	With controls+: Coeff (SE)	Unadjusted: Coeff (SE)	With controls+: Coeff (SE)
*SEN status (ref.=no SEN)*				
Has SEN	0.83 (0.183)***	0.40 (0.218)†	0.69 (0.144)***	0.35 (0.180)†
Has statement of needs	1.14 (0.176)***	0.59 (0.233)*	1.21 (0.160)***	0.70 (0.189)***
*LSLI status (ref.=no LSLI)*				
Has LSLI	0.82 (0.190)***	0.34 (0.214)	0.85 (0.146)***	0.44 (0.162)**

*Notes*: †p < .1; *p < .05; **p < .01; ***p < .001.

+ controls: sex, birth season, ethnic group, housing tenure, parental education, family structure, household employment status (MCS and LSYPE) and parental closeness to child, discipline measures used, child height and weight, maternal depression, prior cognitive ability (MCS) and arguments with parents, parental disability and prior educational attainment (LSYPE). All models adjusted for complex survey design and non-response.

The top panel of Table 3 shows that for younger children, disability is significantly associated with bullying. Focusing on unadjusted differences we see substantially higher risks of being bullied for disabled children compared to non-disabled children. These reflect raw bullying victimisation rates of 8 per cent for non-disabled children, 14 per cent for LSLI, 17 per cent for SEN and 20 per cent for children with a statement. Turning to the fully adjusted models, we see that the increased risk of being bullied is indeed partly accounted for by other risk factors also associated with disability (such as being younger, being a boy, having lower cognitive scores and being obese). Nevertheless, the association remains significant for all disability groups. In line with our prior expectations, the strongest association is found for SEN, with the SEN statement group facing the highest risk.

**Figure 1.**
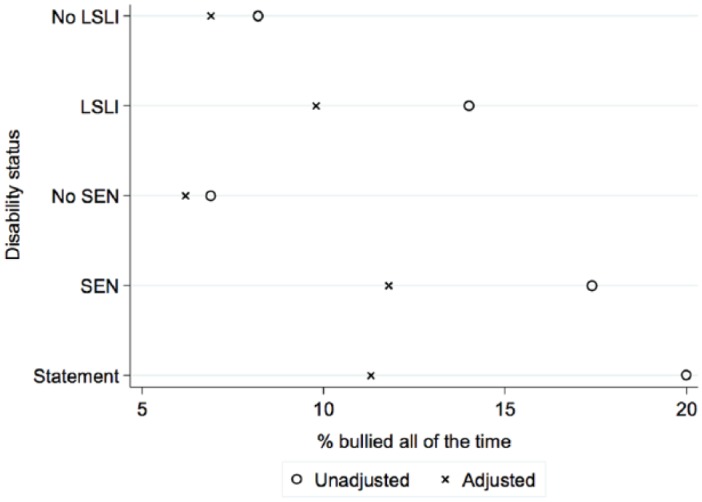
Unadjusted and adjusted estimates of being bullied ‘all the time’ at age seven by disability status. *Source*: Millennium Cohort Study.

These findings are clearly illustrated in Figure 1, which focuses on the estimated chances of being bullied for a child with average characteristics. It shows how factors associated both with disability and bullying substantially reduce the probabilities of being bullied for disabled children, whereas for non-disabled children the probabilities vary little between the adjusted and unadjusted models. Figure 1 shows the increased probability, notwithstanding, of a disabled child being bullied even when all other characteristics are taken into account. For example, although the probability of being bullied decreases for a child who has a statement (from 20 per cent to 11 per cent) or SEN (from 17 per cent to 12 per cent) after all controls are included, their probability of being bullied is still twice that of an ‘average’ child with no SEN (6%).

At age 15, we were able to separate out more complex measures of bullying that were also highly sex-specific: girls are less likely to be subject to physical bullying but more likely to experience relational bullying than boys. The bottom panel of Table 3 clearly shows that at age 15 both SEN and LSLI are associated with frequent physical as well as relational bullying. For physical bullying, the raw rates were around 4 per cent (non-disabled children), 8 per cent (LSLI), 7 per cent (SEN) and 9 per cent (statement). Figures for relational bullying were around 6 per cent (non-disabled children), 13 per cent (LSLI), 10 per cent (SEN) and 16 per cent (statement). However, much of the enhanced likelihood of physical bullying is accounted for by factors associated with disability that also increase the chances of being physically bullied (such as being a boy, having lower educational attainment and having a disabled mother). The introduction of controls reduces the coefficient for disability status on physical bullying by half or more, and renders it non-statistically significant for LSLI and barely significant for SEN at conventional levels. However, children with a statement retain a significantly and substantially higher risk of physical victimisation when compared with otherwise similar non-disabled children.

**Figure 2.**
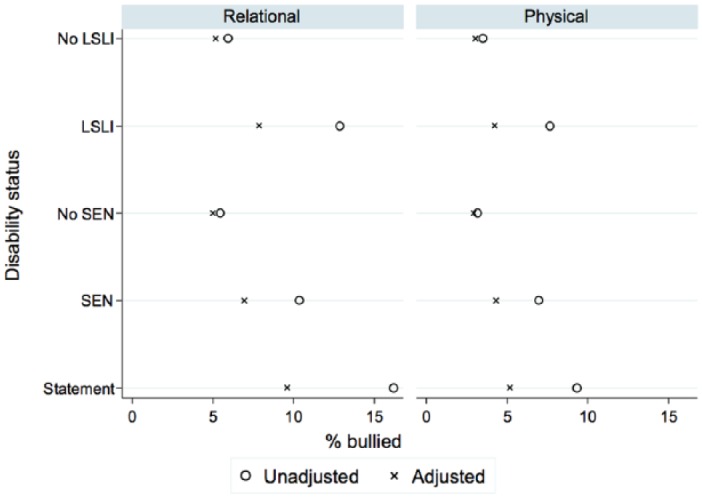
Unadjusted and adjusted estimates of relational and physical bullying at age 15 by disability status. *Source*: LSYPE.

The introduction of controls reduces the coefficients for disability on relational bullying somewhat less. Table 3 shows that both children with a statement or with LSLI have a significantly increased risk of relational bullying victimisation when compared with observationally similar non-disabled children. This emphasises the way in which children may use forms of exclusion and verbal rather than physical intimidation to isolate those who are regarded as ‘different’, at an age when peer conformity is becoming ever more important (Abrams, 2010). The increased risk of relational bullying victimisation by disability status is illustrated in Figure 2.

While consistent with the results at age seven, the age 15 findings are perhaps even more striking. First, when children are older and victimisation rates are generally lower than for younger children, we might expect some of the specific risk associated with the ‘otherness’ of disability to have dissipated. Second, if, as the literature shows, early bullying impacts on social relations and educational attainment, we might have expected some of the impact of the earlier bullying that these children are likely to have experienced to have been reflected in our controls for family conflict and educational attainment. Yet the association between bullying and disability is net of these impacts and remains large and significant. Third, although we found clearer associations for relational rather than physical bullying, there is still evidence for increased risks for physical bullying for children with a SEN statement.

## Conclusions

Recent longitudinal research has established that early bullying experiences have a strong negative impact on social and psychological later life outcomes, over and above the influence of other risk factors such as parental socio-economic background (Arseneault et al., 2010; Takizawa et al., 2014; Wolke et al., 2013). It is therefore pertinent to identify the groups that face a higher risk of being bullied and to consider subsequently the role of early peer victimisation in their life trajectories and outcomes. This study focused on disabled children, a group that has been largely neglected both in bullying as well as life-course research (Powell, 2003; Watson, 2012). Taking into account that earlier qualitative research has suggested that bullying is a common experience among disabled children (Connors and Stalker, 2002; Watson et al., 1999), we sought to document the prevalence of bullying among disabled children and adolescents in England. Acknowledging that disability is socially constructed and that stigma and bullying result from asymmetric relationships rather than personal attributes (Goffman, 1963), our analysis examined the extent to which victimisation of disabled groups was related to their construction as disabled or was accounted for by other characteristics that are also linked to reduced social status and popularity within school networks and which are more prevalent among disabled children (Faris and Felmlee, 2014).

Our analysis confirmed that disabled children and young people in England are facing ‘double disadvantage’ comprising *both* limiting contexts and greater socio-economic disadvantage associated with disability, *and*, additionally, increased risks of bullying and its adverse consequences, during critical periods in their school careers and development. Using a stringent threshold for bullying both in childhood and adolescence, we found that higher victimisation rates of disabled children are partly explained by other risk factors such as age within school year, sex and cognitive ability/educational attainment. Hence, the greater disadvantage that disabled children face in relation to socio-economic disadvantage at home, as well as (linked) family stressors, such as parental disability, also put them at increased risk of being bullied. Moreover, disabled children and young people’s additional challenges in terms of educational development and more conflictual family relationships also increase their vulnerability. To this extent, any reductions in school bullying are likely to enhance the disabled young people’s transitions and adult outcomes.

However, disabled children and adolescents still remained at higher risk of being bullied, net of this wide range of factors, corroborating earlier qualitative and school-based studies (Connors and Stalker, 2002; Sentenac et al., 2013; Watson et al., 1999). We found that disability measured as both SEN and LSLI is implicated in higher risks of being bullied, indicating that the vulnerability of disabled children in the school context is not only a result of institutional labelling. Our prior expectations that effects would be stronger for children with a statement were also confirmed. Our findings therefore provide support for earlier work problematising the practice of labelling for the development of positive disabled identities (Holt, 2004), and draw attention to the school context as a potential site of reproduction of social inequalities. In the context of recent reforms relating to SEN and child disability, future research would benefit from assessing the extent to which current SEN policies and practices support appropriate life-course transitions of children with special learning needs (Priestley, 2003).

Life-course research focusing on childhood disability remains scarce (Powell, 2003; Watson, 2012). However, the few existing studies suggest that individuals who experience childhood disability are likely to lag behind across a number of psycho-social dimensions in adulthood (Janus, 2009; Wells et al., 2003). Our study provides large-scale evidence for a process that disability scholars have previously referred to as ‘psycho-emotional disablism’ (Connors and Stalker, 2007; Thomas, 1999), which may be a critical mechanism leading to adverse outcomes among disabled people (Link and Phelan, 2001). By demonstrating that there are specific disability-related bullying risks, we provide additional support for earlier claims that disability should be considered as a factor contributing to the production and reproduction of stratification *in its own right*, independently of factors such as socio-economic status (Jenkins, 1991). By providing representative evidence on the victimisation of disabled children and young people, we underline the importance of furthering understanding of the victimisation of this group and draw attention to the school context as another site of reproduction of disability-related inequality. Overall, our study emphasises the importance of incorporating the role of bullying into future studies focusing on the outcomes of childhood disability and within theoretical accounts on the ways disabilities are constructed.

## Notes

1. Full tables are available from authors upon request.
